# Case report: clinical and postmortem findings in four cows with rib fracture

**DOI:** 10.1186/s13104-017-2415-1

**Published:** 2017-02-06

**Authors:** Ueli Braun, Sonja Warislohner, Udo Hetzel, Karl Nuss

**Affiliations:** 1 Department of Farm Animals, Vetsuisse-Faculty, University of Zurich, Winterthurerstrasse 260, 8057 Zurich, Switzerland; 2 Institute of Veterinary Pathology, Vetsuisse Faculty, University of Zurich, Zurich, Switzerland

**Keywords:** Case report, Cattle, Rib, Fracture, Clinical findings, Postmortem findings

## Abstract

**Background:**

Published reports of rib fractures in adult cattle are limited to the occurrence of chronic rib swellings caused by calluses, which are unremarkable from a clinical standpoint, whereas studies identifying clinical signs of rib fractures were not found in a literature search. This report describes the clinical and postmortem findings in four cows with rib fractures.

**Case presentation:**

The 13th rib was fractured in three cows and the 11th rib in the remaining cow; three fractures were on the right and one on the left side. Clinical and postmortem findings varied considerably, and percussion of the rib cage elicited a pain response in only one cow. One cow had generalised peritonitis because of perforation of the rumen by the fractured rib. One cow was recumbent because of pain and became a downer cow, and two other cows had bronchopneumonia, which was a sequel to osteomyelitis of the fracture site in one. In the absence of a history of trauma, the diagnosis of rib fracture based on clinical signs alone is difficult.

**Conclusions:**

Although rib fractures undoubtedly are very painful, the four cases described in this report suggest that they are difficult to diagnose in cattle because associated clinical signs are nonspecific.

## Background

Typical clinical sings of rib fracture are difficult to characterise in cattle even though rib fractures occur commonly in this species. It can be assumed that this is a painful condition similar to rib fracture in people. The idea for the study came after the first author slipped on a patch of ice and sustained an extremely painful rib fracture. In calves, the most common cause of rib fracture is trauma incurred during forced extraction during dystocia [1–10]. Causes of rib fracture in mature cattle include trauma sustained in falls, horn injuries, collisions with objects or other animals, mishaps during transport, mounting by other cattle and entrapment [11]. Spontaneous rib fracture has been described in cattle with phosphorus deficiency [12]. Acute rib fracture can be diagnosed by palpation or percussion, which elicits a pronounced pain response, whereas old fractures are recognised by swelling over the affected rib due to ossification [11]. Osseous sequestration is rare after rib fracture [13]. Swellings at the costochondral junctions of the 7th to 9th ribs indicating previous fractures are often seen in lame cows [14, 15], and mid-shaft rib fractures of the 2nd to 4th ribs have been associated with a low body condition score, particularly in older cows [16]. A study of approximately 2000 cows from 13 dairy herds showed that the herd prevalence of palpable swellings at the costocondral junctions of the 7th to 9th ribs was 3.6–26.8% with 15.8% of cows affected overall [15, 17]. Those findings were supported by the results of a study from the United Kingdom [18]. The prevalence of rib swellings was 5.0% in cows with a mobility score of 0 (not lame) and 47.2% in cows with a score of 3 (distinct lameness), and it was postulated that rib fractures were the result of lame cows dropping to the ground faster than sound cows when lying down [15]. This leads to compression of the chest and fracture at the point of the greatest curvature, which is the costochondral junction of the 7th to 9th ribs. Examination of the internal aspects of more than 300 slaughter cows revealed ossified swellings approximately 10–20 cm dorsal to the costochondral junction of the 3rd to 5th ribs in about 10% of the carcases [19]. Studies on clinical signs and management of rib fractures were not identified in a literature search using the PubMed and VetMed Resource databases from 1975 to 2015 and the keywords, *cattle*, *rib* and *fracture*. The purpose of this report was to describe 4 cases of rib fracture in cows seen at our clinic from 1991 to 2015 (Tables 1, 2). These cases emphasize that clinical signs of rib fracture vary widely in cows and antemortem diagnosis is very difficult.

**Table 1:** Clinical and postmortem findings in four cows with rib fractures

Cow No.	Rectal temperature (°C), heart and respiratory rates (per minute)	Main clinical signs	Clinical diagnosis	Main postmortem findings
1	39.4/80/30	Rumen atony, positive foreign body tests, abdominal guarding, loss of negative abdominal pressure, abdominocentesis yielded an exudate	Peritonitis	Fracture of left 13th rib, perforation of rumen, generalised peritonitis
2	39.7/72/52	Tachypnoea, increased breath sounds, positive foreign body tests, percussion of right thoracic wall painful, epistaxis, terminal recumbency	Bronchopneumonia	Fracture of 13th thoracic vertebra and right 13th rib, purulent osteomyelitis with extension to lung, lung abscess
3	38.3/68/28	Downer cow postpartum initially because of hypocalcaemia and hypomagnesaemia and then because of pain caused by rib fracture	Downer cow syndrome	Fracture of right 11th rib with acute haemorrhage
4	39.3/96/52	Tachypnoea, increased breath sounds, pleuritic friction rubs, cough, epistaxis, pleural effusion, terminal recumbency	Bronchopneumonia	Acute splintered fracture of right 13th rib with soft tissue laceration, severe acute bronchopneumonia caused by *Mannheimia haemolytica*, coxarthrosis and rupture of the iliofemoral ligament on the left side

**Table 2:** Results of haematological analysis in four cows with rib fractures

Cow No.	Variable (reference range)				
	Haematocrit (33–36%)	Total leukocyte count (5000–10,000/µl)	Plasma protein (60–80 g/l)	Fibrinogen (3–5 g/l)	Glutaraldehyde test (10–15 mins)
1	42	7400	61	6	ND
2	26	9200	92	12	2.5
3	40	8500	62	6	>10
4	35	9900	78	8	>10

*ND* not done

## Case presentation

Cow 1: A 3-year-old Brown Swiss cow that had calved without assistance six weeks previously was referred to our clinic because of mild ruminal tympany, lack of response to treatment with a magnet and a 3-day history of anorexia. The cow was in poor general health and had a rectal temperature of 39.3 °C, a heart rate of 80 beats per minute (bpm), a respiratory rate of 30 breaths per minute and no rumen motility. There was bruxism and guarding of the abdominal wall. Tests for foreign bodies (withers pinch, percussion of the reticular region and lateral thorax, upward pressure applied to the xyphoid region using a pole) were positive. Transrectal examination showed that there was no negative pressure in the abdomen. Radiographs of the reticulum were normal, and haematological analysis showed haemoconcentration. Retroperitoneal fluid collected by abdominocentesis was opaque and yellow and had a specific gravity of 1026, a protein concentration of 33 g/l and 10,500 leukocytes/µl (70% neutrophils, 29% monocytes and 1% lymphocytes). A diagnosis of peritonitis possibly caused by a perforating abomasal ulcer was made, and because of a poor prognosis, the cow was slaughtered. There was generalised peritonitis with widespread adhesions throughout the abdomen, particularly on the left side of the rumen, and the abdominal cavity contained a large amount of fibrinous exudate. The last rib on the left side was fractured, and a sharp bone fragment pointing inward had perforated the rumen, resulting in leakage of rumen contents and subsequent peritonitis.

Cow 2: A 3.5-year-old Brown Swiss cow that was 6 months pregnant was referred to our clinic because of stiff gait and reluctance to lie down, which had been observed for three months before referral. The cow had a normal appetite but appeared painful and uncomfortable particularly when the rumen was full. The general health of the cow was mildly abnormal, and the rectal temperature was 39.7 °C, the heart rate 72 bpm and the respiratory rate 52 breaths per minute. Respiration was costoabdominal but had a strong abdominal component, and breath sounds were increased. Rumen motility was strong and rumen fill and stratification were normal. Foreign body tests were consistently positive, abdominal guarding was present, and percussion of the right thoracic wall elicited a painful response. Haematological analysis showed anaemia and increased protein and fibrinogen concentrations, and the clotting time in the glutaraldehyde test was shorter than normal. Ultrasonography of the thorax and abdomen revealed no abnormalities, and radiography of the reticulum and lungs showed an increase in the radiodensity of the interstitial lung tissue. Endoscopy of the upper respiratory tract was normal. A tentative diagnosis of bronchopneumonia was made and the cow treated with danofloxacin (1.25 mg/kg, intravenously; Advocid, Zoetis, Switzerland) and 10 l of a solution containing 9 g NaCl and 50 g glucose per litre via an indwelling jugular vein catheter, daily, for three days. On the fourth day, the cow was unable to rise and was euthanased with pentobarbital (70 mg/kg, intravenously; Streuli Pharma, Uznach, Switzerland) immediately after blood and purulent material were seen flowing from both nostrils accompanied by severe tachypnoea during an attempt to lift the cow. Postmortem examination showed a fracture of the 13th thoracic vertebra and 13th rib on the right side and purulent osteomyelitis that had extended into the right lung with subsequent abscess formation.

**Fig. 1:**
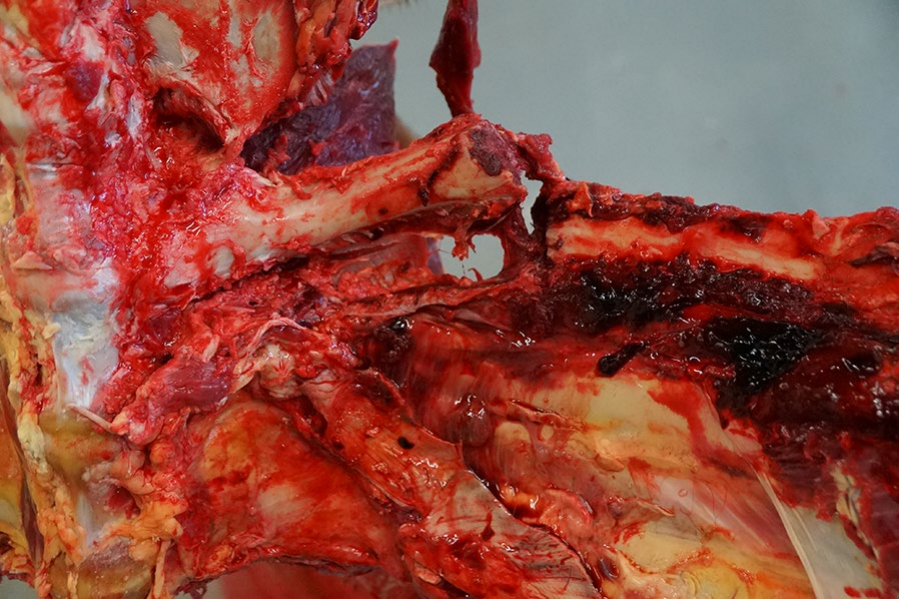
Postmortem view of a rib fracture. Postmortem view of a fracture of the 11th rib on the right side in cow 3

Cow 3: A 12-year-old Brown Swiss cow was referred to our clinic because of inability to rise after an unassisted calving, despite multiple treatments with calcium borogluconate administered intravenously and sodium phosphate given orally. The cow was bright and alert and in sternal recumbency at the time of admission. The general health of the cow was only mildly affected, the appetite was reduced, the rectal temperature was 38.3 °C, the heart rate was 68 bpm and the respiratory rate was 28 breaths per minute. The cow had ruminal atony and passed small amounts of faeces. Haematological analysis showed haemoconcentration, hypocalcaemia (1.95 mmol/l, normal 2.3–2.6 mmol/l), hypomagnesaemia (0.50 mmol/l, normal 0.8–1.0 mmol/l) and a mild increase in the activity of creatine kinase (4773 U/l, normal 70–170 U/l). A diagnosis of downer cow syndrome associated with hypocalcaemia and hypomagnesaemia was made and the cow was treated daily for 5 days with 10 l of a solution containing 9 g NaCl and 50 g glucose per litre, 500 ml of a 40% calcium borogluconate solution (Calcamyl-40 M, Graeub, Bern, Switzerland) and 500 ml of glucose solution containing magnesium (Glucamagnesium, Werner Stricker, Zollikofen, Switzerland), via an indwelling jugular vein catheter, with no response. The cow also received multiple doses of calcium (Calcivet, Provet, Lyssach, Switzerland) and magnesium preparations (Magnoral, Streuli Pharma) administered via an orogastric tube, and ketoprofen (3 mg/kg; Rifen, Streuli Pharma) intravenously. The cow was rolled from one side onto the other every four hours and several attempts to lift the cow using a transport sling were made every day; however, the cow failed to support any weight despite the fact that serum electrolyte concentrations had normalised. Likewise, the cow did not support any body weight in a flotation tank (Aqua Cow Rise System, Denmark) and was therefore euthanased (same method as used in cow 2) after 5 days of unsuccessful treatment. Postmortem examination showed a fracture of the 11th rib on the right side and associated haemorrhage, about 15 cm distal to the vertebral column (Fig. 1). The fracture was just caudal to the diaphragm and there were adhesions between the lungs and the parietal pleura and diaphragm in that area.

Cow 4: A 9-year-old Holstein–Friesian cow was referred to our clinic for evaluation of anorexia. Five days before referral, the cow had been chased by another cow and had become trapped between a wall and a post. Strong traction was required to free the cow and for a short time afterwards, she was unable to rise. The referring veterinarian treated the cow with enrofloxacin, flunixin meglumine and dexamethasone. The general health of the cow was moderately abnormal, and physical examination revealed sunken eyes, reduced skin turgor, a rectal temperature of 39.3 °C, a heart rate of 96 bpm and a respiratory rate of 52 breaths per minute. The cow stood with her head and neck extended because of dyspnea, which was characterised by a marked abdominal component of respiration. There was bilateral seromucous nasal discharge and a spontaneous cough, and auscultation of the chest revealed increased breaths sounds and a pleuritic friction rub. The stride in the hind legs was shorter than normal and a cracking sound originating from the hind end of the cow was audible at the walk. The blood concentrations of calcium, phosphorus and magnesium were normal, and the activity of creatine kinase was mildly elevated (1355 U/l). Ultrasonograms of the thorax and abdomen showed bilateral pleural effusion, which had a depth of about 2 cm. The right lung was consolidated and suspended in the pleuritic fluid and the pleura had several comet-tail artifacts. The ventral lung regions were radiodense. Endoscopy of the trachea to the level of the bifurcation revealed white respiratory secretion, from which *Mannheimia haemolytica* was isolated. A diagnosis of bronchopneumonia attributable to *Mannheimia haemolytica* was made and the cow treated with cefquinom (2.5 mg/kg; Cobactan 7.5%, MSD Animal Health, Luzern, Switzerland), administered subcutaneously, and 10 l of a solution containing 9 g NaCl and 50 g glucose per l and calcium borogluconate (Calcamyl-40MP), administered intravenously via an indwelling jugular vein catheter. The condition of the cow deteriorated despite treatment, and euthanasia was elected because she could not stand and was groaning continuously. Postmortem examination showed an acute splintered fracture of the last rib on the right about 15 cm distal to the costovertebral joint accompanied by acute haemorrhage and soft tissue lacerations. The cow also had severe acute fibrinous and seropurulent bronchopneumonia because of infection with *Mannheimia haemolytica* and coxarthrosis with rupture of the iliofemoral ligament of the left hind limb.

## Discussion

Of 20,100 cattle with internal diseases examined at the Department of Farm Animals, University of Zurich, between 1991 and 2015, only four had clinical signs of a rib fracture. Thus, rib fracture had an incidence of 0.0005% over a 25-year period and can be considered an extremely rare cause of referral in cattle. Rib fractures are extremely painful in people, and it can be assumed that the same is true for cattle. In contrast, old rib fractures, which are apparent as rib swellings and not associated with clinical signs, are common in cattle with chronic lameness and some herds have a prevalence of about 16% [15]. The discrepancy between the low incidence of cases with clinical signs in our study and the relatively high prevalence of healed rib fractures reported in the literature is likely because clinical signs of acute rib fracture in cattle often are nonspecific and not associated with visible and palpable signs. In contrast, inspiratory dyspnoea is a typical sign of rib fracture associated with callus formation at the fracture site in calves [10]. In the cases described in this report, clinical signs varied depending on the circumstances of the injury that led to the fracture. In cow 1, perforation of the rumen by the fractured rib resulted in leakage of ingesta into the abdominal cavity and subsequent generalised peritonitis. Cow 3 had downer cow syndrome and acute haemorrhage at the fracture site, and it was assumed that the cow fell because of hypocalcaemia thereby sustaining a rib fracture. Rib swellings seen in lame cows indicating previous rib fractures also have been attributed to falls [14, 15, 18]. In the two remaining cows, the rib fracture was accompanied by acute bronchopneumonia. In cow 2, this was the direct result of osteomyelitis with spread of purulent material into the lungs. In the remaining cow, there was no obvious association between acute bronchopneumonia caused by *Mannheimia haemolytica* infection and the fracture of the last rib. However, two other cows from the same herd with bronchopneumonia caused by the same agent had been seen at our clinic a few weeks previously. It is therefore conceivable that the cow had a latent infection and the stress associated with the entrapment and rib fracture led to bronchopneumonia.

We are aware of only one reference that described the clinical signs of cattle with acute rib fractures; a distinct pain response, characterised by grunting, is elicited by percussion of or pressure applied to the rib cage [11]. Although our examination protocol includes percussion of the rib cage using a rubber mallet—in addition to foreign body tests (withers pinch, percussion of the reticular region, pressure applied to the xyphoid region)—a pain response to percussion of the rib cage could be elicited only in cow 2.

Likewise, the results of haematological analysis were not specific for rib fracture. Two cows had haemoconcentration, which was attributed to generalised peritonitis (cow 1) and dehydration (cow 3). The increased protein and fibrinogen concentrations and the abnormal glutaraldehyde test result in cow 3 reflected inflammation attributable to osteomyelitis and lung abscessation.

The likely cause of the rib fracture was a fall associated with hypocalcaemia in cow 3 and entrapment in cow 4. In cows 1 and 2, the cause was not clear but injury caused by a fall or inflicted by the horns of herd mates was possible. Cattle with horn injuries are often seen at our clinic [20, 21]. The 13th rib was fractured in three cows and the 11th rib in the remaining cow, and three fractures were on the right and one on the left side. Other authors reported that ribs 9–11 are most commonly affected because they form the greatest curvature of the trunk and absorb the primary impact during a fall [15]. This report was limited to the description of cows with rib fracture; it was not possible to establish valid diagnostic guidelines because of the wide variation in clinical signs and post-mortem findings in the affected cows. This underscores the importance of careful history taking (with respect to possible trauma and the presence of horned herd mates) and thorough clinical examination.

## Conclusions

Although rib fractures undoubtedly are very painful, they are difficult to diagnose in cattle because associated clinical signs are nonspecific. Cows with splintered rib fracture, perforation of the rumen by the fractured rib and other complications including osteomyelitis, abscess formation and recumbency should be euthanased rather than treated. The occurrence of rib fracture can be minimised by installing non-slip barn floors, eliminating bottlenecks where cows become trapped and injured and by dehorning aggressive cows.
